# Ultra-sensitive RDT performance and antigen dynamics in a high-transmission *Plasmodium falciparum* setting in Mali

**DOI:** 10.1186/s12936-020-03389-0

**Published:** 2020-09-03

**Authors:** Emily N. Reichert, Jen C. C. Hume, Issaka Sagara, Sara A. Healy, Mahamadoun H. Assadou, Merepen A. Guindo, Rebecca Barney, Andy Rashid, Ihn Kyung Yang, Allison Golden, Gonzalo J. Domingo, Patrick E. Duffy, Hannah C. Slater

**Affiliations:** 1 Diagnostics Program, PATH, Seattle, WA USA; 2 Laboratory of Malaria Immunology and Vaccinology, National Institute of Allergy and Infectious Diseases, National Institutes of Health, Rockville, MD USA; 3 Malaria Research and Training Center, Mali-National Institute of Allergy and Infectious Diseases International Center for Excellence in Research, University of Science, Techniques and Technologies of Bamako, Bamako, Mali

**Keywords:** Malaria, Ultra-sensitive RDT, Antigenemia, HRP2, pLDH

## Abstract

**Background:**

The recent expansion of tools designed to accurately quantify malaria parasite-produced antigens has enabled us to evaluate the performance of rapid diagnostic tests (RDTs) as a function of the antigens they detect—typically histidine rich protein 2 (HRP2) or lactate dehydrogenase (LDH).

**Methods:**

For this analysis, whole blood specimens from a longitudinal study in Bancoumana, Mali were used to evaluate the performance of the ultra-sensitive HRP2-based Alere™ Malaria Ag P.f RDT (uRDT). The samples were collected as part of a transmission-blocking vaccine trial in a high transmission region for *Plasmodium falciparum* malaria. Furthermore, antigen dynamics after successful anti-malarial drug treatment were evaluated in these samples using the Q-Plex Human Malaria Array (4-Plex) to quantify antigen concentrations.

**Results:**

The uRDT had a 50% probability of a positive result at 207 pg/mL HRP2 [95% credible interval (CrI) 160–268]. Individuals with symptomatic infection remained positive by uRDT for a median of 33 days [95% confidence interval (CI) 28–47] post anti-malarial drug treatment. Biphasic exponential decay models accurately captured the population level post-treatment dynamics of both HRP2 and *Plasmodium* LDH (pLDH), with the latter decaying more rapidly. Motivated by these differences in rates of decay, a novel algorithm that used HRP2:pLDH ratios to predict if an individual had active versus recently cleared *P. falciparum* infection was developed. The algorithm had 77.5% accuracy in correctly classifying antigen-positive individuals as those with and without active infection.

**Conclusions:**

These results characterize the performance of the ultra-sensitive RDT and demonstrate the potential for emerging antigen-quantifying technologies in the field of malaria diagnostics to be helpful tools in distinguishing between active versus recently cleared malaria infections.

## Background

Malaria is most commonly diagnosed in humans by either microscopy or rapid diagnostic tests (RDTs), which detect antigens produced by malaria parasites [1, 2]. The antigens histidine rich protein 2 (HRP2) and lactate dehydrogenase (LDH) are those most commonly targeted by RDTs. HRP2 antigen is expressed only by *Plasmodium falciparum* malaria, and RDTs targeting *P. falciparum* infections typically detect HRP2, while LDH is a constitutive enzyme expressed by all *Plasmodium* species. RDTs can specifically detect *P. falciparum* infections through antibodies targeting subspecies epitopes on the LDH antigen, as well as all *Plasmodium* species through conserved epitopes. Of note, HRP2-based RDTs can exhibit cross-reactivity with HRP3 due to antigenic similarity.

Until recently, malaria RDT evaluation programmes have focused on benchmarking malaria diagnostic tests against parasite density measured either by microscopy or nucleic acid tests, although antigen concentrations of standard samples have recently begun to be included [3–5]. RDTs have been considered to perform equivalently to microscopy in terms of sensitivity and specificity [6], with a recognition that HRP2-based RDTs may provide false positives due to the long half-life of circulating HRP2 [1, 2]. The advent of new RDTs, such as the ultra-sensitive HRP2-based Alere™ Malaria Ag P.f RDT (Abbott, South Korea) with a tenfold lower limit of detection for HRP2 than previous RDTs [7, 8] has incentivized the research community to better understand antigen dynamics in infected populations [8–12]. Concurrently, new assays for antigen quantification have been developed both as research tools on the Luminex platform [13, 14] and as the commercially available Q-Plex™ Human Malaria Array (Quansys Biosciences, USA) [9].

This report describes the performance of the ultra-sensitive Alere Malaria Ag P.f RDT (uRDT) on samples collected in a phase 1 clinical trial malaria vaccine study in Bancoumana, Mali. This trial assessed Pfs25M-EPA/Alhydrogel^®^ and Pfs230-EPA/Alhydrogel^®^ individually and in combination for safety and functional immunogenicity in malaria-exposed Malian adults (ClinicalTrials.gov: NCT02334462). The vaccine was given on a 0, 1, 6 month schedule in the 1st year with a booster dose 12 months after dose 3. Given the longitudinal nature of the study cohort, the population level antigen dynamics for both HRP2 and LDH were investigated, as well as relative antigen abundance post-treatment, as determined with the Q-Plex enzyme-linked immunosorbent assay (ELISA).

## Methods

### Study design and data collection

Individuals in this analysis were part of a double-blind, randomized, phase 1 clinical trial conducted by the Laboratory of Malaria Immunology and Vaccinology (LMIV)/National Institute of Allergy and Infectious Diseases (NIAID)/National Institutes of Health (NIH). Bancoumana is a rural village 60 km from Bamako, Mali, with high prevalence of *Plasmodium falciparum.* This trial investigated the safety and immunogenicity of Pfs230D1M-EPA/Alhydrogel^®^ and Pfs25M-EPA/Alhydrogel, both transmission-blocking vaccines against *P. falciparum*. Transmission-blocking vaccines were administered on study days 0, 28, 169, and 540. Blood smears were prepared before each vaccination, at least monthly post vaccination, or when clinically indicated. Starting 1 week after the third and fourth vaccinations, blood smears were prepared twice a week for 6 weeks at the same time that subjects underwent direct skin feeding assays with colony-raised *Anopheles coluzzii* to assess malaria parasite transmission. Whole blood samples to be analysed in this study were collected at per-protocol scheduled blood draws 1-6 weeks after the fourth vaccination (study days 547, 554, 568, and 582) during peak malaria transmission season (July–December). Between 1 and 21 mL of whole blood was collected at each blood draw for each study participant, all who agreed to have blood samples stored for future research prior to enrollment. Individuals who at any point presented with symptomatic malaria, defined as any parasitaemia by blood smear or RDT positive result with symptoms consistent with malaria, were treated with anti-malarial drugs artemether–lumefantrine (Coartem or Laritem) for uncomplicated malaria and artemether for severe malaria. Whole blood samples for individuals treated with anti-malarials were collected at the same per-protocol frequency as the remainder of the study cohort. Microscopy-positive asymptomatic individuals were not treated, per Malian National Policy on Malaria Control Guidelines.

### Sample evaluation

Frozen whole blood samples (n = 622) were sent to PATH’s laboratory (Seattle, WA, USA) for further evaluation. Two ultra-sensitive HRP2-based Alere Malaria Ag P.f RDTs (uRDT), product number 05FK140, lots 05LDB005A and 05LDB004A, were used to test in duplicate each specimen, all of which had been stored at − 80 °C. The test required 5 µL of whole blood and was run following the standard workflow outlined in Das et al. [15]. A final uRDT result was generated from duplicate uRDT results in agreement only; when results were either discordant or invalid, results were considered not confirmed and excluded from final analyses.

HRP2 and *Plasmodium* LDH (pLDH) concentrations were quantified using the Q-Plex Human Malaria Array (4-Plex), which quantifies pLDH by detecting pan epitope [9]. Standards of recombinant protein with known antigen concentration are run on each plate allowing quantification through standard curves. Ranges of quantification for HRP2 and pLDH were 1.07–16,500 pg/mL and 14.41–525,700 pg/mL, respectively. For numeric analyses, samples with antigen concentrations beyond the limit of quantification (LOQ) for Q-Plex were treated as (upper LOQ) * 2 and (lower LOQ)/2. Thresholds above which samples were defined as antigen positive, determined through receiver operating characteristics analysis to identify the optimal sensitivity and specificity tradeoff, were 2.30 pg/mL for HRP2 and 47.8 pg/mL for pLDH [9]. Parasite count by microscopy included both gametocytes and asexual parasites and was estimated as parasites per 1000 white blood cells (WBCs), but is reported in parasites/µL, using the conversion of 8000 WBCs/µL [16]. Gametocyte counts were combined with asexual parasite counts as both have been shown to express HRP2 and LDH [17].

### Statistical analysis

Data compilation and statistical analysis was performed using R 3.6.0 software [18]. A Bayesian logistic regression model with study level random effects was used to model the relationship between HRP2 concentration and probability of detection by uRDT. A log10 transformation was applied to the HRP2 concentration data and a Gaussian distribution with a mean of zero and standard deviation of three was used for the prior. Four chains of 1000 iterations were ran after a burn-in of 500 iterations, from which median predictions and 95% Bayesian credible intervals (CrI) were taken. A Kaplan–Meier survival curve was generated to estimate probability of a uRDT-positive result for individuals post successful anti-malarial treatment. HRP2 and pLDH dynamics post-treatment were modelled by fitting monophasic and biphasic exponential decay models. A monophasic decay assumes a constant decay rate over time, whereas the biphasic decay model allows for two different decay rates, typically a rapid initial decay followed by a period of slower decay. The functional forms for these two models are:

$${\text{Monophasic: }}\log_{10} \left( {concentration} \right) = k_{1} t + C_{0}$$

$${\text{Biphasic: }}\log_{10} \left( {concentration} \right) = \left\{ {\begin{array}{*{20}l} {k_{1} t} & {\textrm{if}\; t < t_{switch} } \\ {k_{1} t + k_{2} \left( {t - t_{switch} } \right)} & {\textrm{if} \;t \ge t_{switch} } \\ \end{array} } \right\} + C_{0}$$

where $t$ = time (in days), $k_{1} , k_{2}$ are decay parameters, $t_{switch}$ is the switch point between “fast” and “slow” decay, and C_0_ is the log10 initial concentration. An individual level random effect was incorporated into each model, accounting for individual variation in antigen concentration at time of treatment (t = 0) and therefore fitting unique values of C_0_ to each individual. Models were compared using ANOVA and those that minimized both Akaike information criterion (AIC) and Bayesian information criterion (BIC) were ultimately selected. Predictive intervals were obtained by using the predictInterval function in the R package merTools, which estimates the distribution of all model parameters while incorporating uncertainty in both fixed and random effects. This function was run over 1000 simulations to obtain 95% predictive intervals. Finally, receiver operating characteristic (ROC) curves were calculated to determine optimal thresholds for predicting active versus recently cleared *P. falciparum* infection, with thresholds maximizing Youden’s index (the sum of sensitivity and specificity) defined as optimal.

## Results

### Study population

Blood samples were collected from 160 adults between the ages of 18–53 years living in Bancoumana or the surrounding area. Collections of interest were performed between September–November 2017, totaling 622 blood specimens. Mean participant age was 40.7 years [standard deviation (SD) 8.4] at time of blood draw visits. Out of 160 participants, 110 [69%; 95% confidence interval (CI); 61–76] were positive by ultra-sensitive rapid diagnostic test (uRDT) and 99 (62%; 54%–69%) were positive by microscopy at least once during the sampling window. Furthermore, 101 (63%; 55%–71%) of these participants presented with symptomatic infection at least once. Of the 166 microscopy-positive whole blood specimens, 155 (93%) were *P. falciparum* only, 3 (1.8%) were *P. falciparum* mixed infections, and 8 (4.8%) were non-*P. falciparum (Plasmodium ovale* or *Plasmodium malariae)*. Non-*P. falciparum* infections as identified by microscopy were excluded, leaving 614/622 (98.7%) of specimens for further evaluation.

### Performance of the ultra-sensitive RDT

**Fig. 1:**
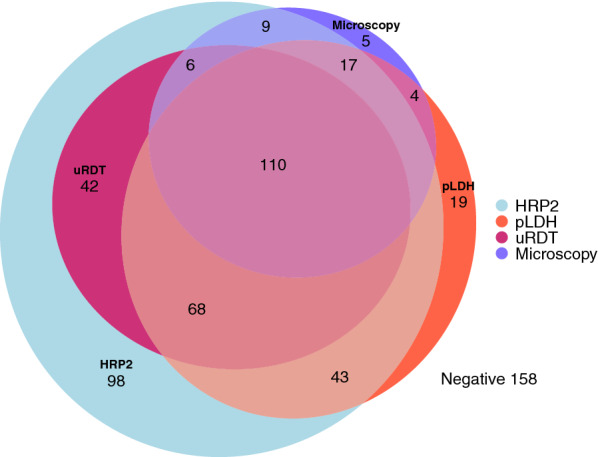
Relationship between various methods of detection. Venn diagram visualizing overlap in detection between microscopy and new generation diagnostics (uRDT and Q-Plex ELISA) for the 579/614 blood samples with conclusive results for all four detection methods. HRP2, histidine rich protein 2; pLDH, *Plasmodium* lactate dehydrogenase; uRDT, ultra-sensitive rapid diagnostic test

The performance of the uRDT was evaluated against microscopy as well as HRP2 and all-malaria pLDH confirmed by Q-Plex (Table 1). There were ten samples (1.6%) with discordant uRDT results excluded from uRDT evaluation results [geometric mean (GM): 190 pg/mL HRP2]. All uRDT-positive results were also HRP2-positive. Only 51% of uRDT-positive infections were microscopy positive, whereas 73% of microscopy-positive infections were positive by uRDT (Fig. 1). The uRDT detected 80.7% of infections that were both HRP2 and microscopy-positive versus 56.1% HRP2-positive only, indicating a higher sensitivity performance for high-density infections. There were five microscopy-confirmed infections that were not confirmed by either HRP2, pLDH, or the uRDT, all of which had low parasitaemia (< 16 parasites/µL). Only two-thirds (66.2%) of pLDH-positive samples also had sufficient HRP2 to be detected by uRDT.

**Table 1:** Performance of the uRDT against Q-Plex ELISA and microscopy detection

	HRP2 Q-Plex ELISA reference, % (95% CI)	pLDH Q-Plex ELISA reference, % (95% CI)	Microscopy reference, % (95% CI)
Sensitivity	58% (53–63)	68% (63–74)	78% (71–84)
Specificity	100% (100–100)	84% (80–88)	74% (70–78)
PPV	100% (100–100)	78% (73–83)	51% (45–57)
NPV	53% (48–58)	77% (72–81)	90% (87–93)

Results are given with associated 95% CIs. Whole blood samples were defined as HRP2- and pLDH-positive with concentrations > 2.30 pg/mL and > 47.8 pg/mL, respectively. Microscopy-positive samples were those with any *P. falciparum* parasites detected per 1000 WBCs

*ELISA* enzyme-linked immunosorbent assay, *LDH* lactate dehydrogenase, *PPV* positive predicted value, *NPV* negative predicted value

**Fig. 2:**
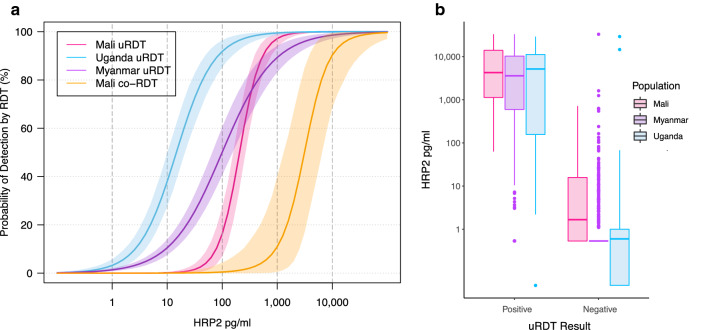
Estimated probability of uRDT detection by HRP2 concentration. **a** Fitted relationship between uRDT performance (probability of detection) and HRP2 concentration for Mali (n = 614, high transmission) and two other sample sites: Uganda (n = 607, high transmission) and Myanmar (n = 1847, low transmission) using a Bayesian logistic regression model. Probability of detection is also shown for a small number (n = 35) of specimens with a co-RDT run in the field in Mali (orange). Shaded regions show 95% credible intervals for median model predictions. **b** Boxplot of HRP2 distributions in Mali, Myanmar, and Uganda study cohorts stratified by uRDT result. *co-RDT* conventional RDT, *HRP2* histidine rich protein 2, *uRDT* ultra-sensitive rapid diagnostic test

Comparable uRDT positivity and HRP2 concentration data exists for two recently published studies conducted in asymptomatic individuals, one in Uganda (n = 607, high transmission) [7], and one in Myanmar (n = 1847, low transmission) [8]. Details of these studies have been previously published and are briefly summarized in Additional file 1. Figure 2a shows the probability of detection by uRDT in relation to HRP2 concentration for Mali in comparison to the studies conducted in Uganda and Myanmar [7, 8]. Differences emerged in uRDT detection limits among the three study populations: there was a 50% probability of testing positive by uRDT at HRP2 thresholds of 207 pg/mL [95% credible interval (CrI) 160–268] in Mali, 15 pg/mL (11–21) in Uganda, and 101 pg/mL (66–156) in Myanmar. The established limit of detection (LOD) for the commercial Alere Malaria Ag P.f uRDT is 80–100 pg/mL, per laboratory evaluation by Das et al. [15]. The HRP2 distributions for samples in each study population stratified by uRDT result are visualized in Fig. 2b.

Compared to a small subset of samples in Mali for which conventional RDTs (co-RDTs) were run in the field (n = 35), the uRDT was on average more than tenfold more sensitive in its LOD than the co-RDT: 50% probability of detection was achieved at 207 pg/mL HRP2 for the uRDT compared to 3140 pg/mL HRP2 for co-RDT. However, both tests had higher detection thresholds than expected (80–100 pg/mL for uRDT and 800 pg/mL for co-RDT) [15]. Out of 415 HRP2-positive samples, 104 (25%) were between the calculated 50% detection threshold of the uRDT and co-RDT, with 37/104 (36%) of these also microscopy positive for *P. falciparum.*

### Antigenaemia, detection, and treatment status

In Mali, the geometric mean for HRP2 was 55.4 pg/mL (geometric SD 49.7) and for pLDH was 101.8 pg/mL (geometric SD 17.7). The correlation for the transformed log10 value of each antigen (pLDH and HRP2) with log10 parasitaemia by microscopy was poor, but was higher for pLDH than HRP2 (R^2^ = 0.61 and R^2^ = 0.29, respectively) among all microscopy-positive samples (Additional file 1: Figures S1 and S2).

**Fig. 3:**
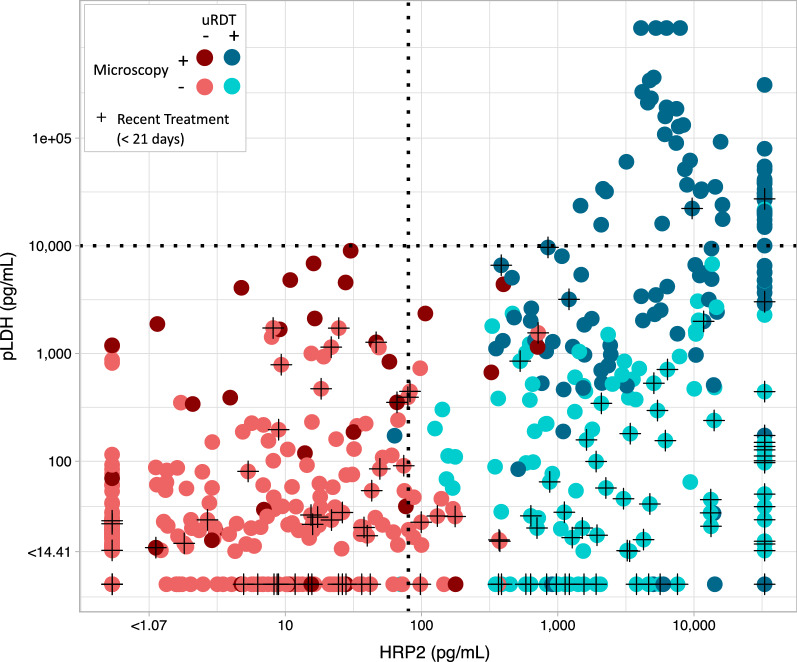
Classification of diagnostic performance by relative biomarker concentrations. Each blood sample with results for microscopy, Q-plex, and uRDT (n = 579) is represented as a single point colored by uRDT and microscopy results. Dotted lines indicate the most sensitive thresholds for currently available RDTs: ~ 10,000 pg/mL for pLDH, an estimate based on detection of ~ 200 parasites/µL [9, 30, 31], and 80–100 pg/mL for HRP2 [15]. Additionally, black crosses indicate those with recent antimalarial drug treatment (previous 21 days). HRP2, histidine rich protein 2; pLDH, *Plasmodium* lactate dehydrogenase; RDT, rapid diagnostic test; uRDT, ultra-sensitive rapid diagnostic test

Figure 3 shows the HRP2 and pLDH concentrations for each sample in the Mali study classified by both uRDT and microscopy result. Geometric mean (GM) HRP2 concentration was on average 4002 pg/mL for uRDT-positive samples and 3.2 pg/mL for uRDT-negative samples. Microscopy-positive samples also had higher concentrations of HRP2 (GM: 1430 pg/mL, compared to 18 pg/mL for those microscopy-negative). Of interest, a majority (63%) of microscopy-positive samples had < 10,000 pg/mL pLDH, below the estimated LOD for currently available pLDH-based diagnostic tests. Of those with pLDH > 10,000 pg/mL, only one sample had HRP2 < 100 pg/mL. A combined HRP2, pLDH diagnostic without improved sensitivity for pLDH would, therefore, not have captured a significantly higher number of infected individuals than the HRP2-based test alone in this high transmission *P. falciparum* setting.

**Fig. 4:**
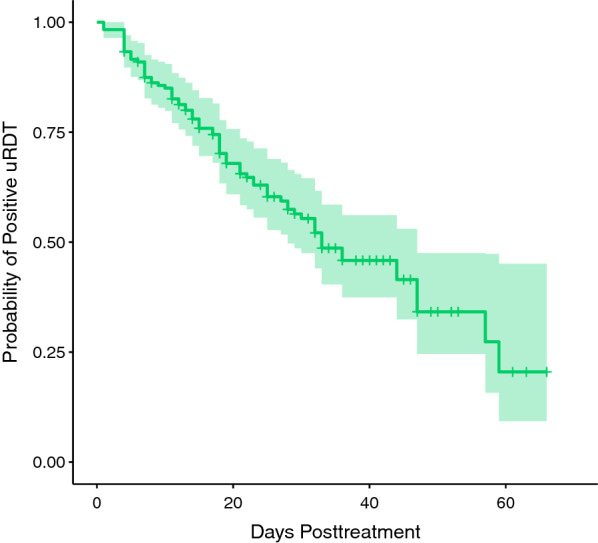
Persistence in uRDT detection post-treatment. A Kaplan–Meier survival curve showing the probability of remaining uRDT positive following successful treatment for symptomatic *P. falciparum* infection. The shaded region represents the median model’s 95% CI. Median time to uRDT negativity was 33 days (95% CI 28–47), with day of antimalarial drug treatment for symptomatic infection defined as day 0. Samples with evidence of unsuccessful treatment or recurrent infection, defined as those positive for *P. falciparum* by microscopy at any point after 1 day post-treatment, were excluded. *uRDT* ultra-sensitive rapid diagnostic test

Individuals who at any point in this study presented with symptomatic malaria were treated with anti-malarial drugs. There were 130 symptomatic infections treated in 101 unique individuals. Of samples from “recently treated” individuals, defined as receiving anti-malarial drugs in the previous 21 days, a majority (52.5%) were still positive by uRDT, with the highest proportion (43.3%) uRDT-positive and microscopy-negative (Fig. 3). Recently treated samples have considerably lower values of pLDH (GM: 50 pg/mL) compared to samples from individuals that have not recently been treated (GM: 156 pg/mL). The opposite is true for HRP2 concentration (367 pg/mL compared to 91 pg/mL). A Kaplan–Meier survival curve fitted to samples from individuals up to 70 days post-treatment (n = 187) with no evidence of persistent *P. falciparum* infection by microscopy (i.e. no microscopy-positive result at any timepoint after 1 day post-treatment) estimated that median time to uRDT negativity was 33 days post-treatment (95% CI 28–47) (Fig. 4).

### Antigen dynamics post-treatment

**Fig. 5:**
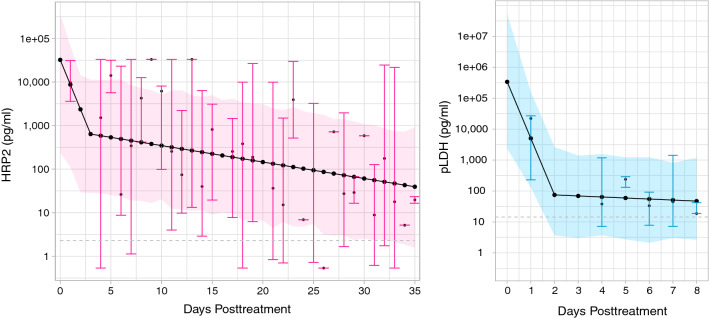
Biphasic exponential decay models for HRP2 and pLDH post-treatment. Fitted population-level biphasic decay models, with a switch time (knot) of 3 days post-treatment for HRP2 (pink) and 2 days post-treatment for pLDH (blue). Black dots indicate median model predictions, with shaded regions showing associated 95% prediction intervals. Median antigen concentrations with 95% CIs, depicted by pink and blue vertical lines, are also plotted for each day data were available post-treatment. Horizontal grey lines indicate the lower LOQ for each antigen—values below the LOQ were set to half this LOQ and retained in the analyses. Samples with evidence of recurrent *P. falciparum* parasitemia by microscopy at any point in the follow-up window after 1 day post-treatment were excluded. HRP2, histidine rich protein 2; pLDH, *Plasmodium* lactate dehydrogenase

Monophasic and biphasic exponential decay models with individual-level random effect were fitted to HRP2 and pLDH data for samples with no microscopic evidence of recurrent *P. falciparum* post-treatment. Models were fit up to 35 days post-treatment for HRP2 (152 samples, 75 individuals) and only 8 days for pLDH (39 samples, 37 individuals) to avoid uncertainty in model predictions once median antigen values declined below the limit of quantification. Using criterion that minimized both AIC and BIC, a biphasic exponential model best estimated population level antigen decay (Fig. 5) and was a significantly better fit compared to monophasic for both HRP2 and pLDH (P = 0.02 and P = 0.003, respectively). The optimal switch point (knot) was 2 days for pLDH and 3 days for HRP2. Decay parameters for pLDH were k_1_ = − 1.83 and k_2_ = 1.80 compared to k_1_ = − 0.57 and k_2_ = 0.53 for HRP2; pLDH initially decayed more rapidly than HRP2, as is consistent with the literature [19]. Based on median model predictions, the average time to reach “undetectable” levels of < 100 pg/mL was 3 days for pLDH compared to 26 days for HRP2 among this study cohort. It is important to note that fitted median antigenaemia at time of treatment was fairly low compared to other studies (380,000 pg/mL pLDH and 28,000 pg/mL HRP2) [19], potentially because participants were being treated quickly as soon as symptomatic infection presented due to frequent study visits.

**Fig. 6:**
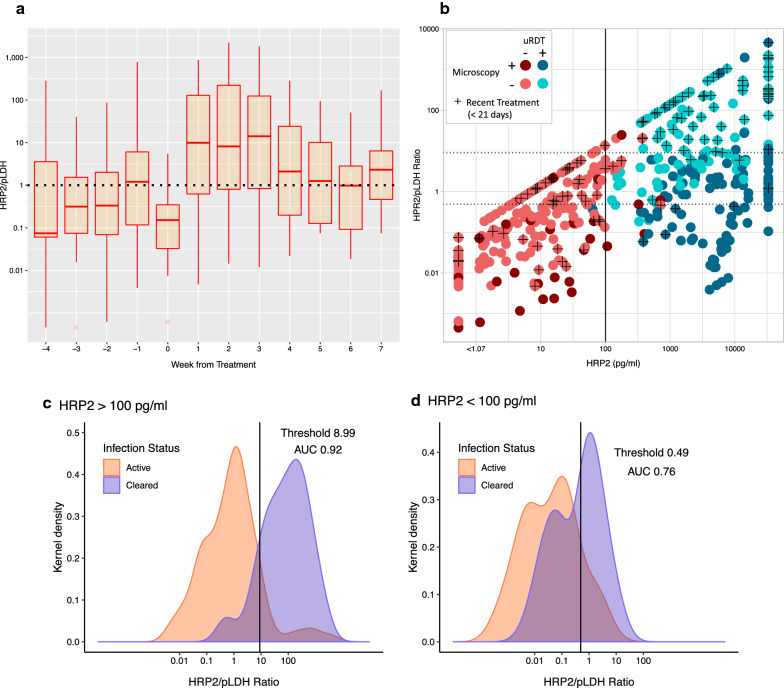
HRP2:pLDH ratios as tool to predict infection status. **a** Boxplot of HRP2:pLDH ratios for individuals within 4 weeks pretreatment to 7 weeks post-treatment (n = 311). **b** Scatterplot of the relationship between HRP2:pLDH vs. HRP2. **c** Density plot of HRP2:pLDH ratios for individuals with active vs. cleared *P. falciparum* infection and HRP2 > 100 pg/mL. With a cutoff of 8.99 HRP2:pLDH ratio, cleared infection is predicted with sensitivity = 88.9%, specificity = 89.7%, and AUC = 0.92. **d** Density plot of HRP2:pLDH ratios for individuals with active vs. cleared infection and HRP2 ≤ 100 pg/mL. With a cutoff of 0.49 HRP2/pLDH ratio, cleared infection is predicted with sensitivity = 64.1%, specificity = 85.7%, and AUC = 0.77. AUC, area under the curve; HRP2, histidine rich protein 2; pLDH, *Plasmodium* lactate dehydrogenase

Differing initial rates of decay between HRP2 and pLDH post-treatment indicated the ratio of these antigens’ concentrations may differ for recently treated individuals. Using only samples within 4 weeks pretreatment to 7 weeks post-treatment (311 samples from 89 individuals), HRP2:pLDH ratios were calculated. Samples from six study participants treated twice during this time frame were included as separate samples with differing days of treatment. The median HRP2:pLDH ratio was 0.348 [interquartile range (IQR): 0.07–2.11] pretreatment and 3.41 (IQR: 0.28–51.0) post-treatment (Fig. 6a). HRP2:pLDH ratios were significantly elevated at weeks 1–3 post-treatment, so “recent treatment” was, therefore, defined as treatment with anti-malarial drugs within the past 21 days. Motivated by this difference, attempts were made to distinguish recently cleared but antigen-positive *P. falciparum* infection from active infection based on the HRP2:pLDH ratio. Active infections were defined as those positive for *P. falciparum* by microscopy, whereas cleared infections were defined as microscopy-negative but recently treated (< 21 days ago) with persistent antigenaemia (HRP2 and/or pLDH positive). The relationship between the ratio, HRP2 concentration, and recent treatment history is visualised in (Fig. 6b). Recently treated individuals with successful clearance of *P. falciparum* parasitaemia appeared clustered above different threshold ratios over and under 100 pg/mL HRP2, as confirmed by ROC curve analysis. A pair of values (for HRP2 and HRP2:pLDH) were then selected such that sensitivity and specificity were maximized. If HRP2 is > 100 pg/mL, the optimal threshold (HRP2:pLDH ratio) for classifying whether samples come from individuals with cleared infection is 8.99, above which cleared infection due to recent anti-malarial treatment is predicted [area under the curve (AUC): 0.92] (Fig. 6c). Here the sensitivity is 88.9% and specificity is 89.7%. If HRP2 is ≤ 100 pg/mL, the optimal threshold is 0.49 HRP2:pLDH (AUC: 0.77), here sensitivity = 64.1% and specificity = 85.7% (Fig. 6d). Overall, this classification algorithm performed with 77.5% sensitivity and 88.9% specificity (AUC: 0.83).

Finally, there were 11/614 samples (1.8%) from eight unique individuals positive by direct skin feeding (DSF) experiments. On average, the HRP2:pLDH ratio was lower (P = 0.02) for individuals with a positive DSF result in reference to microscopy and DSF negative samples (see Additional file 1: Table S1). The small number of individuals positive by DSF, a potential result of transmission-blocking vaccines administered prior to blood draw, limited the extent of our direct skin feeding analysis.

## Discussion

This analysis uses data available from both laboratory and field testing in Bancoumana, Mali, to inform three overarching objectives, to: (1) evaluate performance of the uRDT compared to other diagnostic methods, (2) investigate how relative antigen concentrations can classify infections, and (3) better understand the post-treatment dynamics of pLDH and HRP2.

Comparing the HRP2 threshold at which there was a 50% probability of detection by uRDT with the same values from two other studies in Uganda and Myanmar [7, 8] resulted in unexpected differences. HRP2 concentrations in Uganda were quantified using a Bi-Plex Human Malaria Array, an earlier version of the Q-Plex ELISA with a lower LOD (0.1 pg/mL) [7], potentially contributing to observed differences in detection thresholds. Other sources of variation could be (but were not confirmed) lot-to-lot variation in performance of the uRDT, variability in class of HRP2 present at the different locations [20], storage conditions of tests, and/or interpretation of test results. Overall, results indicate a need for further evaluation of the uRDT LOD in the field based on antigenaemia, similar to the large-scale systematic review of co-RDT detection by parasitaemia [21].

In this Malian population, pLDH had a stronger correlation with parasitaemia than HRP2, with the constraint that parasitaemia was quantified by microscopy and not quantitative polymerase chain reaction (qPCR). This is consistent with findings that residual HRP2 lingers after parasite clearance, whereas pLDH has a shorter half-life and is more indicative of active infection [22, 23]. This analysis is important in the context of future development or adoption of pLDH-based assays to address emerging *pfhrp2/3* deletions [25, 26]. In this dataset, there were 12 microscopy-positive, HRP2-negative cases, 4 of which were confirmed to be non-falciparum infections. Of the remaining eight cases, all had low parasite densities (≤ 20 parasites/mL) and only three had a significant pLDH signal (> 300 pg/mL). Further molecular analysis is required to confirm if these are *pfhrp2/3* deletions [24, 25].

Several first-order kinetics models have previously been used to fit HRP2 dynamics [22, 26]. Here biphasic exponential decay models were found to best capture pLDH and HRP2 clearance post-treatment [19]. The nature of biphasic exponential decay (fast, then slow decay) means that previous models may overestimate antigen concentrations initially in the days following treatment [27].

One of the concerns accompanying introduction of the uRDT is that due to HRP2 persistence, ultra-sensitive HRP2-based diagnostics may lead to overtreatment due to individuals with recently cleared infections testing positive and being treated with anti-malarial drugs when there may be another infection or illness causing fever [28]. The ability to use a patient’s antigen concentrations to predict if they are in a stage of typical antigen decline post-treatment would be beneficial both to avoid unnecessary retreatment with anti-malarial drugs and to better understand levels of active infection in the population. This need to distinguish between previous versus active infection led us to develop a novel algorithm for distinguishing recently cleared infections from active ones based on both HRP2 concentration and HRP2:pLDH ratios. Although pLDH alone can be a reliable indicator of active infection, it can be difficult to classify pLDH-positive infections as active versus recently cleared without detailed drug treatment histories. Therefore, although perhaps not viable as a standard case management tool, our approach could be used for routine monitoring of drug efficacy at sentinel surveillance sites and to improve estimates of prevalence in cross-sectional surveys. Overall sensitivity of the classification algorithm was promising (77.5%), with predictive power highest for samples with > 100 pg/mL HRP2. In our analysis, HRP2 and pLDH levels below the LOQ were treated as LOQ/2, although most samples likely cleared pLDH within the 21-day post-treatment window due its more rapid clearance dynamics. In order for HRP2:pLDH ratios to become a reproducible metric for distinguishing recently cleared *P. falciparum* infections in the future, a standardized protocol for dealing with pLDH values of 0 pg/mL (or < LOQ) in HRP2:pLDH calculations will need to be defined.

This analysis was limited to individuals in a high-transmission *P. falciparum* setting and to adults over 18, whereas children under 5 years carry the majority of the global malaria burden [29]. qPCR data was also not collected in this study. Without being able to account for submicroscopic infections, a significant proportion of the infectious reservoir may be being ignored [21]. Further analyses should incorporate data from low-transmission settings and on low-density, submicroscopic infections.

## Conclusions

A deeper understanding of HRP2 and pLDH antigen dynamics in malaria endemic populations will greatly inform the understanding of the performance of RDTs and the utility of RDTs in malaria interventions beyond case management. The data presented here, only considering the relative proportions of two malaria antigens (HRP2 and LDH), suggests that antigen dynamics can be used to differentiate active infections from recently treated infections in *P. falciparum* cases. Further studies analysing a range of longitudinal datasets that include anti-malarial drug history are required to improve upon and validate this approach.

## Supplementary information

**Additional file 1: Figure S1.** Scatterplot showing correlation between parasite count by microscopy and HRP2 concentration quantified by Q-Plex ELISA (R^2^ = 0.29) for all samples *P. falciparum* positive by microscopy (> 0 parasites/µL). Abbreviations: ELISA, enzyme-linked immunosorbent assay; HRP2, histidine rich protein 2; pLDH, *Plasmodium* lactate dehydrogenase. **Figure S2.** Scatterplot showing correlation between parasite count by microscopy and pLDH concentration quantified by Q-Plex ELISA (R^2^ = 0.61) for all samples *P. falciparum* positive by microscopy (> 0 parasites/µL). **Table S1.** Antigen ratios by direct skin feed (DSF) infectivity status. Geometric means for the two antigens of interest, HRP2 and pLDH, along with the geometric mean HRP2:pLDH ratio for study participants that were DSF-positive (n = 11) or DSF-negative (n = 603), grouped by microscopy result. Individuals positive by DSF had a significantly lower HRP2:pLDH ratio (p = 0.02) on average in reference to DSF and microscopy negative individuals. Abbreviations: geo, geometric; HRP2, histidine rich protein 2; pLDH, Plasmodium lactate dehydrogenase; SD, standard deviation.

## Data Availability

The datasets used and/or analyzed during the current study are available from the corresponding author on reasonable request. R code for all analyses and figures are publicly available at https://github.com/PATH-Global-Health/Reichert_Mali_antigens.

## Abbreviations

AIC: Akaike information criterion
BIC: Bayesian information criterion
CI: Confidence interval
GM: Geometric mean
HRP2: Histidine rich protein 2
LDH: Lactate dehydrogenase
LMIV: Laboratory of Malaria Immunology and Vaccinology
LOD: Limit of detection
LOQ: Limit of quantification
NIAID: National Institute of Allergy and Infectious Diseases
NIH: National Institutes of Health
pLDH: *Plasmodium* lactate dehydrogenase
RDT: Rapid diagnostic test
ROC: Receiver operating characteristic
uRDT: Ultra-sensitive HRP2-based Alere™ Malaria Ag P.f RDT
SD: Standard deviation
