# Supplementary material for: Ultra-sensitive RDT performance and antigen dynamics in a high-transmission Plasmodium falciparum setting in Mali
Source: Malar J. 2020 Sep 3;19:323. doi: 10.1186/s12936-020-03389-0 (PMC7469912; doi:10.1186/s12936-020-03389-0)
Supplement: Supplementary file 1 — Additional file 1: Figure S1. Scatterplot showing correlation between parasite count by microscopy and HRP2 concentration quantified by Q-Plex ELISA (R2 = 0.29) for all samples P. falciparum positive by microscopy (> 0 parasites/µL). Abbreviations: ELISA, enzyme-linked immunosorbent assay; HRP2, histidine rich protein 2; pLDH, Plasmodium lactate dehydrogenase. Figure S2. Scatterplot showing correlation between parasite count by microscopy and pLDH concentration quantified by Q-Plex ELISA (R2 = 0.61) for all samples P. falciparum positive by microscopy (> 0 parasites/µL). Table S1. Antigen ratios by direct skin feed (DSF) infectivity status. Geometric means for the two antigens of interest, HRP2 and pLDH, along with the geometric mean HRP2:pLDH ratio for study participants that were DSF-positive (n = 11) or DSF-negative (n = 603), grouped by microscopy result. Individuals positive by DSF had a significantly lower HRP2:pLDH ratio (p = 0.02) on average in reference to DSF and microscopy negative individuals. Abbreviations: geo, geometric; HRP2, histidine rich protein 2; pLDH, Plasmodium lactate dehydrogenase; SD, standard deviation. [file 12936_2020_3389_MOESM1_ESM.docx]

**Additional Information**

**Uganda and Myanmar studies additional details**

Data pertaining to the Uganda and Myanmar studies have recently been published^[[1]](#footnote-1)^^[[2]](#footnote-2)^. This next section provides a brief summary of both studies.

**Ethics approvals**

*Uganda:* Specimens from Uganda were collected under a study approved by the University of California San Francisco (UCSF) (IRB No.11-05995), Makerere University (IRB No. 2011-0167), and London School of Hygiene and Tropical Medicine (LSHTM) (IRB No. 5943).

*Myanmar:* The METF project under which the Myanmar data was collected has ethical approval from the Lower Myanmar Department of Medical Research Ethics’ committee (reference 73/ETHICS2014) with additional approval for supplementary control for HRP2 detection approved by the Oxford Tropical Research Ethics Committee (OxTREC 516-17). The study was also favorably reviewed by the Tak Community Advisory Board (TCAB-09/REV/2016).

**Recruitment**

*Uganda:* Children and care takers with no recent history of malaria and presenting with no malaria symptoms were recruited.

*Myanmar:* Only adults (>18 years of age) were recruited.

In both studies only individuals with no current malaria symptoms nor recent history of malaria were eligible to participate.

**Figure S1.** Scatterplot showing correlation between parasite count by microscopy and HRP2 concentration quantified by Q-Plex ELISA (R^2^ = 0.29) for all samples *P. falciparum* positive by microscopy (> 0 parasites/µL).

Abbreviations: ELISA, enzyme-linked immunosorbent assay; HRP2, histidine rich protein 2.

**Figure S2.** Scatterplot showing correlation between parasite count by microscopy and pLDH concentration quantified by Q-Plex ELISA (R^2^ = 0.61) for all samples *P. falciparum* positive by microscopy (> 0 parasites/µL).

|  | Geometric mean HRP2 (pg/mL)  (geo SD) | Geometric mean pLDH (pg/mL)  (geo SD) | Geometric mean HRP2:pLDH  (geo SD) | p-value |
| --- | --- | --- | --- | --- |
| Direct skin feed positive (n = 11) | 889 (18) | 6,772 (11) | 0.13 (5.1) | 0.02 |
| Direct skin feed negative, microscopy positive (n = 148) | 1,430 (24) | 2,735 (21) | 0.48 (18) | 0.65 |
| Direct skin feed negative, microscopy negative (n = 455) | 18 (33) | 31 (5.1) | 0.55 (20) | *reference* |

**Table S1. Antigen ratios by direct skin feed (DSF) infectivity status.** Geometric means for the two antigens of interest, HRP2 and pLDH, along with the geometric mean HRP2:pLDH ratio for study participants that were DSF-positive (n = 11) or DSF-negative (n = 603), grouped by microscopy result. Individuals positive by DSF had a significantly lower HRP2:pLDH ratio (p = 0.02) on average in reference to DSF and microscopy negative individuals.

Abbreviations: geo, geometric; HRP2, histidine rich protein 2; pLDH, *Plasmodium* lactate dehydrogenase; SD, standard deviation.

1. Das S, Jang IK, Barney B, et al. Performance of a High-Sensitivity Rapid Diagnostic Test for Plasmodium falciparum Malaria in Asymptomatic Individuals from Uganda and Myanmar and Naive Human Challenge Infections. Am J Trop Med Hyg. 2017;97(5):1540-1550. doi:10.4269/ajtmh.17-0245. [↑](#footnote-ref-1)
2. Landier J, Haohankhunnatham W, Das S, et al. Operational Performance of a Plasmodium falciparum Ultrasensitive Rapid Diagnostic Test for Detection of Asymptomatic Infections in Eastern Myanmar. *J Clin Microbiol*. 2018;56(8):e00565-18. doi:10.1128/JCM.00565-18 [↑](#footnote-ref-2)
